# Efficacy and safety of P2X3 receptor antagonists in refractory or unexplained chronic cough: an updated systematic review and meta-analysis

**DOI:** 10.3389/fphar.2026.1911091

**Published:** 2026-09-29

**Authors:** Yu-Tong An, Shu-Ze Tang, Yi Luo, Le Huang, Xiu-Juan Deng

**Affiliations:** 1 Hunan University of Chinese Medicine, Changsha, China; 2 Affiliated Hospital of Hunan Academy of Chinese Medicine, Changsha, China

**Keywords:** chronic disease, cough, meta-analysis, purinergic P2X receptor antagonists, receptors, purinergic P2X3

## Abstract

**Background:**

Refractory chronic cough and unexplained chronic cough are increasingly understood as clinical expressions of cough hypersensitivity. P2X3 receptor antagonists have been developed to reduce ATP-mediated airway sensory nerve activation, but their benefit must be balanced against taste-related adverse events.

**Methods:**

Randomized placebo-controlled trials evaluating oral P2X3 receptor antagonists in adults with refractory or unexplained chronic cough were included. Objective cough frequency and taste-related adverse events were selected as primary efficacy and safety/tolerability outcomes. Secondary outcomes included Leicester Cough Questionnaire (LCQ) total score, cough severity visual analogue scale (VAS), overall adverse events, and discontinuation due to adverse events. Effects were pooled using random-effects inverse-variance models. Sensitivity analyses, publication-bias assessment, and GRADE certainty ratings were performed.

**Results:**

Eleven trial reports involving 3568 participants were included, yielding 13 independent comparisons for objective cough frequency. P2×3 receptor antagonists reduced objective cough frequency compared with placebo (ratio 0.71, 95% CI 0.63–0.79), corresponding to a 29.3% relative reduction. They also improved LCQ total score (MD 1.44, 95% CI 0.78–2.09) and cough severity VAS (MD -12.73 mm, 95% CI -17.10 to −8.36). Taste-related adverse events were increased (RR 7.36, 95% CI 4.84–11.21), as were overall adverse events (RR 1.21, 95% CI 1.07–1.38) and discontinuation due to adverse events (RR 3.62, 95% CI 2.65–4.93). Leave-one-out analyses did not alter the direction of any pooled estimate. Egger and Begg tests suggested small-study effects for objective cough frequency, but not for safety outcomes with sufficient comparisons. The certainty of evidence was rated as moderate for all prespecified outcomes.

**Conclusion:**

P2×3 receptor antagonists were associated with reductions in objective cough frequency and improvements in patient-reported outcomes in refractory or unexplained chronic cough. These benefits should be balanced against increased safety and tolerability concerns, especially taste disturbance, although receptor selectivity appears to attenuate this risk.

**Systematic Review Registration:**

https://www.crd.york.ac.uk/prospero/, identifier CRD420261422476.

## 1 Introduction

Chronic cough is commonly defined as cough lasting more than 8 weeks, with a global adult prevalence of approximately 10% and considerable variation across regions (Song et al., 2015; Morice et al., 2020). In specialist clinics, a substantial proportion of patients continue to cough despite systematic assessment and treatment of common causes such as asthma, eosinophilic bronchitis, gastroesophageal reflux disease, rhinitis, and medication-related cough (Morice et al., 2020; Gibson et al., 2016; Morice et al., 2007). These patients are usually described as having refractory chronic cough (RCC) when cough persists despite treatment of an identifiable condition, or unexplained chronic cough (UCC) when no cause is identified after appropriate evaluation (Morice et al., 2020; Gibson et al., 2016).

The concept of cough hypersensitivity has helped to unify this clinical picture (Morice et al., 2014; Chung et al., 2013). Patients often report laryngeal irritation, allotussia, hypertussia, and cough triggered by innocuous stimuli such as cold air, talking, perfumes, or eating (Morice et al., 2014; Chung et al., 2013). At the neurobiological level, airway sensory nerves can become sensitized, and ATP-gated purinergic signaling through P2X3-containing receptors has emerged as one of the most important peripheral pathways (Mazzone and Undem, 2016; Ford and Undem, 2013). This mechanism is clinically attractive because it sits close to the afferent cough reflex rather than to a single upstream disease label. Taste disturbance is also biologically plausible, because ATP signaling through P2X2/P2X3-containing receptors is central to communication between taste buds and gustatory nerves (Finger et al., 2005; Vandenbeuch et al., 2015). Existing non-P2X3 treatments, including neuromodulators and behavioral cough-suppression therapy, may help selected patients but are limited by variable response, treatment burden, and tolerability (Ryan et al., 2012; Vertigan et al., 2006; Chamberlain Mitchell et al., 2017; Bowen et al., 2018).

Gefapixant, the first extensively studied P2×3 antagonist, confirmed that blocking this pathway could reduce objective cough frequency (Abdulqawi et al., 2015; Smith et al., 2020a; Smith et al., 2020b; Morice et al., 2021; McGarvey et al., 2022; Niimi et al., 2022a; Matera et al., 2024). However, clinical trials showed a consistent signal for taste disturbance, which is biologically plausible because P2X2/3 heterotrimeric receptors are involved in taste signaling (Finger et al., 2005; Vandenbeuch et al., 2015; Abdulqawi et al., 2015). Newer agents, including eliapixant, sivopixant, filapixant, and camlipixant, were designed with greater selectivity for P2X3 homotrimers (Morice et al., 2021; Niimi et al., 2022a; Dicpinigaitis et al., 2023; Friedrich et al., 2023; McGarvey et al., 2023a; Smith et al., 2025a; Smith et al., 2025b). Whether this selectivity preserves antitussive efficacy while improving tolerability is an important clinical question. AF-219 was the original development code for gefapixant, developed initially by Afferent Pharmaceuticals and subsequently by Merck after acquisition.

Previous reviews and network meta-analyses have supported the antitussive potential of P2X3 receptor antagonism, but the evidence base has continued to evolve, particularly with the publication of recent camlipixant trials and newer analyses of receptor selectivity (Ramadan et al., 2023; Zheng et al., 2023; Yamamoto et al., 2024; Yi et al., 2023). Therefore, we conducted an updated systematic review and meta-analysis of randomized placebo-controlled trials to evaluate the efficacy, safety, and tolerability of P2X3 receptor antagonists in adults with RCC or UCC, with a prespecified subgroup comparison according to receptor selectivity. Accordingly, the term selective P2X3 antagonists is used as a pragmatic subgroup label rather than as evidence that individual agents have identical receptor selectivity, potency, pharmacokinetic exposure, or clinical behavior.

## 2 Methods

### 2.1 Study protocol and registration

This review was reported following the Preferred Reporting Items for Systematic Reviews and Meta-Analyses (PRISMA) statement (Page et al., 2021), and the protocol was registered with PROSPERO under the number CRD420261422476.

### 2.2 Search strategy and study selection

An updated electronic search of bibliographic databases and trial registers was performed, including PubMed/MEDLINE, Embase, Web of Science, the Cochrane Library, CNKI, and ClinicalTrials.gov, and was supplemented by manual screening of the references of relevant trials, reviews, and network meta-analyses. The search strategy combined terms for P2X3 receptor antagonism and chronic cough, including ‘P2X3′, ‘gefapixant’, ‘AF-219′, ‘eliapixant’, ‘sivopixant’, ‘filapixant’, ‘camlipixant’, ‘refractory chronic cough’, ‘unexplained chronic cough’, and ‘chronic cough’. The final search update was performed on 1 June 2026.

Records were screened at title/abstract and full-text levels according to the eligibility criteria. Trial reports meeting the inclusion criteria were retrieved in full, and extracted data were checked against the original publications before quantitative synthesis.

### 2.3 Eligibility criteria

Studies were eligible if they met the following PICOS criteria: (1) participants were adults with RCC, UCC, or refractory/unexplained chronic cough as defined by the trial investigators; (2) the intervention was an oral P2X3 receptor antagonist; (3) the comparator was placebo; (4) at least one extractable efficacy, safety, or tolerability outcome was reported; and (5) the study was randomized and placebo-controlled. Non-randomized studies, case reports, healthy-volunteer studies, pharmacokinetic-only studies, and trials not enrolling chronic cough patients were excluded from the meta-analysis.

### 2.4 Data extraction and outcomes

The extracted study characteristics included first author, publication year, country or region, study design, sample size, intervention, comparator, treatment duration, and reported outcomes. The primary efficacy outcome was objective cough frequency, using the trial-defined objective endpoint at the prespecified primary time point (Birring et al., 2008). Because cough-frequency outcomes were generally analyzed on a logarithmic scale, placebo-adjusted percentage changes were converted to log ratios and standard errors when necessary. LCQ was treated as the principal cough-specific quality-of-life measure, and the commonly used minimal important difference of approximately 1.3 points was considered when interpreting patient-perceived benefit (Birring et al., 2003; Raj et al., 2009).

The primary tolerability outcome was taste-related adverse events, including dysgeusia, hypogeusia, ageusia, hypergeusia, taste disorder, or the trial-defined composite of taste disturbance. These taste-related events were interpreted primarily as tolerability concerns rather than as markers of systemic drug toxicity. Secondary outcomes were LCQ total score, cough severity VAS or CS-VAS, overall adverse events, and discontinuation due to adverse events. Cough severity VAS and CS-VAS were considered comparable patient-reported cough-severity scales when reported on, or convertible to, a 0–100 mm metric, with lower scores indicating less severe cough; a 30-mm reduction was considered a conservative clinically meaningful threshold for chronic cough trials (Birring et al., 2008; Birring et al., 2003; Raj et al., 2009). Continuous patient-reported outcomes were extracted as mean differences with standard errors when a treatment difference and confidence interval or standard error was available.

For multi-arm dose-ranging trials, one clinically relevant intervention dose was selected for the main pairwise meta-analysis to maintain independence of the placebo comparison. The preferred dose was the clinically developed or late-phase target dose when available, such as gefapixant 45/50 mg twice daily. When no target dose was established, the dose with the clearest trial-defined efficacy signal or the highest dose with extractable data was used. The selected comparison for each multi-arm or dose-ranging study, together with the rationale for selection, is listed in Table 1. This approach was used to avoid double-counting shared placebo groups and to keep each trial comparison statistically independent in the main pairwise synthesis. For crossover trials, treatment effects reported by the trial authors were used for continuous outcomes, and safety events were summarized according to the treatment exposure reported in each trial.

**TABLE 1:** Selected dose for the main pairwise synthesis and rationale.

Compound	Trial report/comparison	Dose selected in main synthesis	Reason for selection	Reviewer-relevant caveat
AF-219 (gefapixant)	Abdulqawi 2015	600 mg BID	Only active randomized treatment dose with extractable efficacy and tolerability data	Very early crossover evidence; not directly comparable with later selective compounds
Gefapixant	Smith 2020 ERJ dose-escalation studies; Smith 2020 phase 2b; COUGH-1/COUGH-2	50 mg BID in phase 2 studies; 45 mg BID in COUGH-1/COUGH-2	Selected because 45/50 mg BID was the clinically developed dose range and provided extractable efficacy and safety/tolerability data across the gefapixant program	Lower-dose arms were not included in the main pairwise analysis to avoid double-counting placebo groups
Eliapixant	Morice 2021 phase 2a; Dicpinigaitis 2023 PAGANINI	750 mg BID in Morice 2021; 75 mg BID in PAGANINI	Selected according to the trial-defined dose level with extractable treatment effect used for the main efficacy/tolerability synthesis	The two eliapixant reports differed in design, duration, and dose-ranging structure
Sivopixant	Niimi 2022; McGarvey 2023	150 mg QD in Niimi 2022; 300 mg QD in McGarvey 2023	Selected because these were the available or principal extractable active-dose comparisons for the included sivopixant trials	Nominal doses should not be compared directly with BID doses of other compounds
Filapixant	Friedrich 2023	250 mg BID	Selected as the highest dose level with extractable cough-frequency, patient-reported, and tolerability data in the dose-escalation study	Short dose-escalation exposure limits inference about longer-term clinical positioning
Camlipixant	Smith 2025 RELIEF; Smith 2025 SOOTHE	200 mg BID	Selected because this was the highest reported dose and the main extractable dose level used for efficacy and tolerability endpoints in the included camlipixant trials	Late-stage and regulatory interpretation may evolve as full data from ongoing or recently reported programs become available

BID, twice daily; QD, once daily. The table describes the dose selected for the main independent pairwise synthesis only. It does not imply pharmacokinetic or pharmacodynamic equivalence across compounds.

### 2.5 Risk of bias assessment

Risk of bias was assessed using the revised Cochrane risk-of-bias tool for randomized trials (RoB 2), consistent with the Cochrane framework for randomized trial appraisal (Higgins et al., 2011). The assessment considered bias arising from the randomization process, deviations from intended interventions, missing outcome data, outcome measurement, and selection of the reported result. For crossover trials, period effects, carryover effects, and the suitability of within-participant outcome assessment were also considered. Overall judgments were categorized as low risk of bias, some concerns, or high risk of bias.

### 2.6 Statistical analysis

Random-effects models were used as the main analytical approach because the trials differed in antagonist, receptor selectivity, dose, treatment duration, phase of development, and endpoint timing (DerSimonian and Laird, 1986). Log ratios were pooled for objective cough frequency and back-transformed to ratios and relative percentage reductions. Risk ratios (RRs) were pooled for dichotomous safety and tolerability outcomes, with a continuity correction applied when a study contained a 0 cell. Mean differences (MDs) were pooled for LCQ and VAS outcomes. Heterogeneity was assessed using Cochran’s Q and *I* ^*2*^ statistics (Higgins et al., 2003). Subgroup analyses compared less selective P2X3/P2X2/3 antagonists with more selective P2X3 antagonists. Additional exploratory subgroup analyses examined trial phase and treatment duration for objective cough frequency and taste-related adverse events when sufficient data were available.

Sensitivity analyses were performed by sequentially omitting one comparison at a time. Funnel plots were inspected, and small-study effects were evaluated using Egger’s regression test and Begg’s rank correlation test when at least 10 comparisons were available for an outcome (Egger et al., 1997; Begg and Mazumdar, 1994; Sterne et al., 2011). These assessments were interpreted cautiously because funnel-plot asymmetry may also arise from clinical heterogeneity, dose differences, or early-phase study designs. Formal meta-regression by trial phase, treatment duration, or individual compound was not considered reliable because the number of comparisons was limited and these characteristics were closely correlated with drug, dose, and study design; these factors were therefore addressed qualitatively when interpreting heterogeneity (Sterne et al., 2011). The certainty of evidence for each prespecified outcome was assessed using the GRADE approach (Guyatt et al., 2008; Guyatt et al., 2011; Bals et al., 2011).

## 3 Results

### 3.1 Study selection

The database and register searches identified 1057 records, including 1031 records from bibliographic databases and 26 records from ClinicalTrials.gov. After removal of 687 duplicate records, 370 records were screened by title and abstract. Of these, 326 records were excluded, and 44 reports were sought for full-text retrieval. All 44 reports were retrieved and assessed for eligibility. Thirty-three reports were excluded for reasons including reviews, guidelines or editorials, non-randomized controlled trials, populations not meeting RCC/UCC criteria, ineligible interventions or comparators, unavailable extractable efficacy or safety data, and duplicate or secondary reports. Finally, 11 randomized placebo-controlled trial reports were included in the review. Two reports contributed two independent trial comparisons each: the gefapixant dose-escalation publication reported two separate crossover studies, and the phase 3 gefapixant report included COUGH-1 and COUGH-2. Thus, 13 independent comparisons were available for the primary efficacy analysis. The study selection process is shown in Figure 1.

**FIGURE 1:**
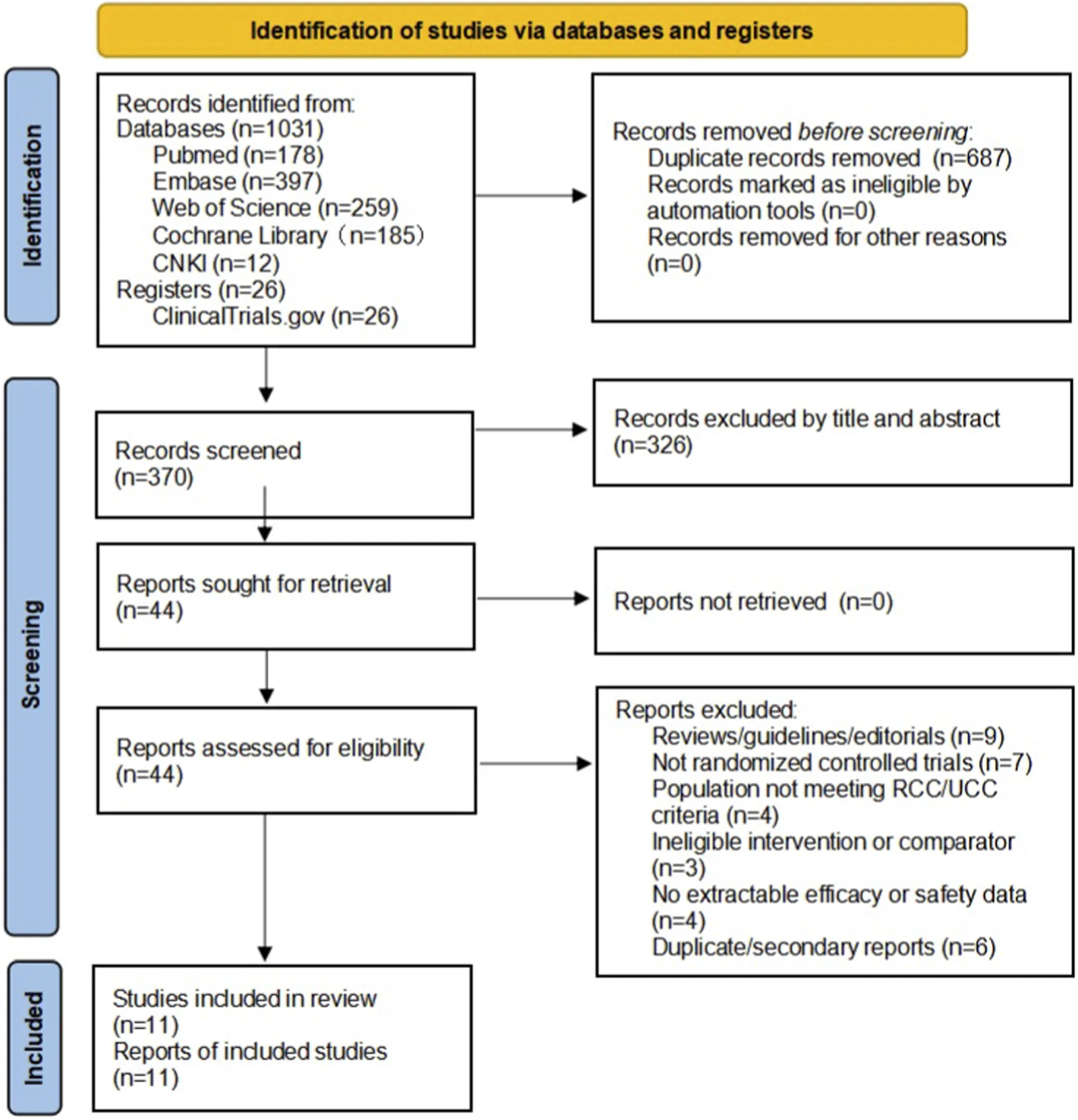
PRISMA flow diagram of study identification, screening, eligibility assessment, and inclusion.

### 3.2 Characteristics of included studies

The 11 included trial reports were published between 2015 and 2025 and included 3568 randomized or treated participants (Abdulqawi et al., 2015; Smith et al., 2020a; Smith et al., 2020b; Morice et al., 2021; McGarvey et al., 2022; Niimi et al., 2022a; Dicpinigaitis et al., 2023; Friedrich et al., 2023; McGarvey et al., 2023a; Smith et al., 2025a; Smith et al., 2025b). Two publications contributed two independent comparisons each: the gefapixant dose-escalation publication reported two separate crossover studies, and the phase 3 gefapixant publication reported COUGH-1 and COUGH-2 separately (Smith et al., 2020a; McGarvey et al., 2022). RELIEF and SOOTHE were published as separate camlipixant trials and were summarized separately because they differed in design and treatment duration (Smith et al., 2025a; Smith et al., 2025b). Trial sample sizes ranged from 24 to 732 participants, and treatment duration ranged from short crossover periods to 52-week long-term exposure. The intervention class included gefapixant, eliapixant, sivopixant, filapixant, and camlipixant. Detailed characteristics of the included studies are summarized in Table 2. The evidence base was not equally developed across individual agents: gefapixant contributed the most mature late-phase evidence, whereas newer selective antagonists were represented by fewer or earlier-stage randomized trials.

**TABLE 2:** Characteristics of included randomized placebo-controlled trial reports.

Study	Country/region	Participants/design	Intervention and comparator	Receptor selectivity	Duration/follow-up	Key outcomes relevant to this review
(Abdulqawi et al., 2015)	United Kingdom	24 adults with refractory chronic cough; single-centre, two-period crossover trial	AF-219 600 mg BID vs. placebo	Less selective P2X3/P2X2/3	Two 2-week treatment periods separated by a 2-week washout	Daytime, 24-h and night-time cough frequency; cough severity and urge-to-cough VAS; CQLQ; AEs including taste disturbance
Smith et al. ERJ (Smith et al., 2020a)	United States of America	59 adults with refractory chronic cough across two crossover dose-escalation studies (study 1, n = 29; study 2, n = 30)	Gefapixant dose escalation: 50/100/150/200 mg BID in study 1 and 7.5/15/30/50 mg BID in study 2 vs. matched placebo	Less selective P2X3/P2X2/3	Two 16-day treatment periods; each dose for 4 days; washout 3–7 days in study 1 and 14–21 days in study 2	Awake, 24-h and night cough frequency; cough severity VAS; cough severity diary; LCQ; AEs including taste disturbance
Smith et al. LRM (Smith et al., 2020b)	United Kingdom and United States of America	253 adults with refractory or unexplained chronic cough; parallel-group phase 2b trial	Gefapixant 7.5, 20, or 50 mg BID vs. placebo	Less selective P2X3/P2X2/3	12 weeks of treatment	Awake, 24-h and sleep cough frequency; cough severity VAS; cough severity diary; LCQ; cough frequency responders; AEs including taste disturbance
(Morice et al., 2021)	United Kingdom specialist centres	40 adults with refractory chronic cough randomized; 37 completed randomized therapy; crossover phase 2a trial	Eliapixant: 10 mg for 1 week, then 50/200/750 mg BID for 1 week per dose level according to sequence vs. placebo	Selective P2X3	Crossover design with placebo and escalating eliapixant periods; follow-up after randomized therapy	24-h cough frequency; awake/asleep cough frequency; cough severity VAS; LCQ; AEs including taste-related AEs
(McGarvey et al., 2022)	Multinational	2044 treated adults with refractory or unexplained chronic cough in two phase 3 trials (COUGH-1, n = 730; COUGH-2, n = 1314)	Gefapixant 15 or 45 mg BID vs. placebo	Less selective P2X3/P2X2/3	COUGH-1: 12-week main period; COUGH-2: 24-week main period; extension periods up to 52 weeks	24-h cough frequency; awake cough frequency; LCQ responder; cough severity diary and VAS; cough frequency responders; AEs including taste disturbance
(Niimi et al., 2022a)	Japan	31 adults with refractory or unexplained chronic cough randomized; multicentre crossover phase 2a trial	Sivopixant 150 mg QD vs. placebo	Selective P2X3	Two 2-week treatment periods separated by a 2- to 3-week washout; 1-week follow-up	Daytime, 24-h, night-time, awake and asleep cough frequency; LCQ; cough severity VAS; EQ-5D-5L; AEs including taste disturbance
(Dicpinigaitis et al., 2023)	Multinational	310 adults with refractory chronic cough randomized at 99 centres in 19 countries; dose-finding phase 2b trial	Eliapixant 25, 75, or 150 mg BID vs. placebo	Selective P2X3	12 weeks of treatment plus 30-day safety follow-up	24-h cough count; awake cough count; cough severity VAS; LCQ; responder endpoints; AEs including dysgeusia and liver safety
(Friedrich et al., 2023)	United Kingdom and Netherlands	23 adults with refractory chronic cough; randomized crossover trial	Filapixant ascending doses: 20, 80, 150, and 250 mg BID vs. placebo	Selective P2X3	Two treatment periods; each dose step for 4 days with 3-day drug-free intervals; 2- to 3-week washout	24-h and awake cough frequency; cough severity VAS; LCQ; cough frequency responders; AEs including taste-related AEs
(McGarvey et al., 2023a)	Europe, United States of America and Japan	406 adults with refractory or unexplained chronic cough randomized; 400 in full analysis set; multicentre phase 2b trial	Sivopixant 50, 150, or 300 mg QD vs. placebo	Selective P2X3	4 weeks of treatment	24-h cough frequency; awake/asleep cough frequency; cough severity VAS; LCQ; PGIC; TEAEs including taste-related TEAEs
(Smith et al., 2025a)	United Kingdom and United States of America	68 adults in safety population; 67 in ITT population; multicentre crossover phase 2a trial	Camlipixant dose escalation: 25, 50, 100, and 200 mg BID vs. placebo	Selective P2X3	Two 16-day treatment periods; each dose for 4 days; 10- to 14-day washout	Awake and 24-h cough frequency; cough severity VAS; LCQ; AEs including taste disturbance
(Smith et al., 2025b)	United States of America, Canada and Europe	310 adults randomized; main population n = 249 and exploratory population n = 61; phase 2b trial	Camlipixant 12.5, 50, or 200 mg BID vs. placebo	Selective P2X3	16-day placebo run-in followed by 4 weeks of randomized treatment	24-h cough frequency; awake and night-time cough frequency; cough severity VAS; LCQ; 30%, 50% and 70% cough frequency responders; TEAEs including taste disturbance

BID, twice daily; QD, once daily; AE, adverse event; TEAE, treatment-emergent adverse event; LCQ, leicester cough questionnaire; VAS, visual analogue scale; CQLQ, Cough-specific Quality of Life Questionnaire; PGIC, Patient Global Impression of Change. N denotes randomized or treated participants reported in each trial report; for crossover trials, N denotes participants entering the randomized treatment sequence rather than the number of treatment exposures. Smith et al., 2020 ERJ, included two separate crossover dose-escalation studies, and McGarvey et al., 2022 included COUGH-1, and COUGH-2; these were treated as independent comparisons in the meta-analysis. Receptor selectivity was classified according to the pharmacological profile described in the original trial reports and related drug-development literature. For dose-ranging trials, one clinically relevant comparison was selected for the main pairwise synthesis to avoid double-counting placebo groups. The main pairwise comparisons selected for quantitative synthesis were AF-219, 600 mg BID, gefapixant 50 mg BID, in phase 2/dose-escalation studies, gefapixant 45 mg BID, in COUGH-1/COUGH-2, eliapixant 750 mg BID, in the crossover phase 2a study, sivopixant 150 mg QD, filapixant 250 mg BID, and camlipixant 200 mg BID, when multiple active doses were reported.

### 3.3 Risk of bias assessment

Risk of bias was assessed using the Cochrane RoB 2 framework. Most parallel-group trials were double-blind and placebo-controlled, with prespecified efficacy outcomes and generally adequate outcome measurement. The main concerns arose from small crossover trials, dose-escalation designs, possible period or carryover effects, differential discontinuation related to taste disturbance, and early termination of RELIEF during the COVID-19 pandemic. Overall, most comparisons were judged as having low risk of bias or some concerns, and no comparison was excluded from quantitative synthesis solely on the basis of risk-of-bias assessment. Detailed judgments are summarized in the risk-of-bias graph (Figures 2 and 3).

**FIGURE 2:**
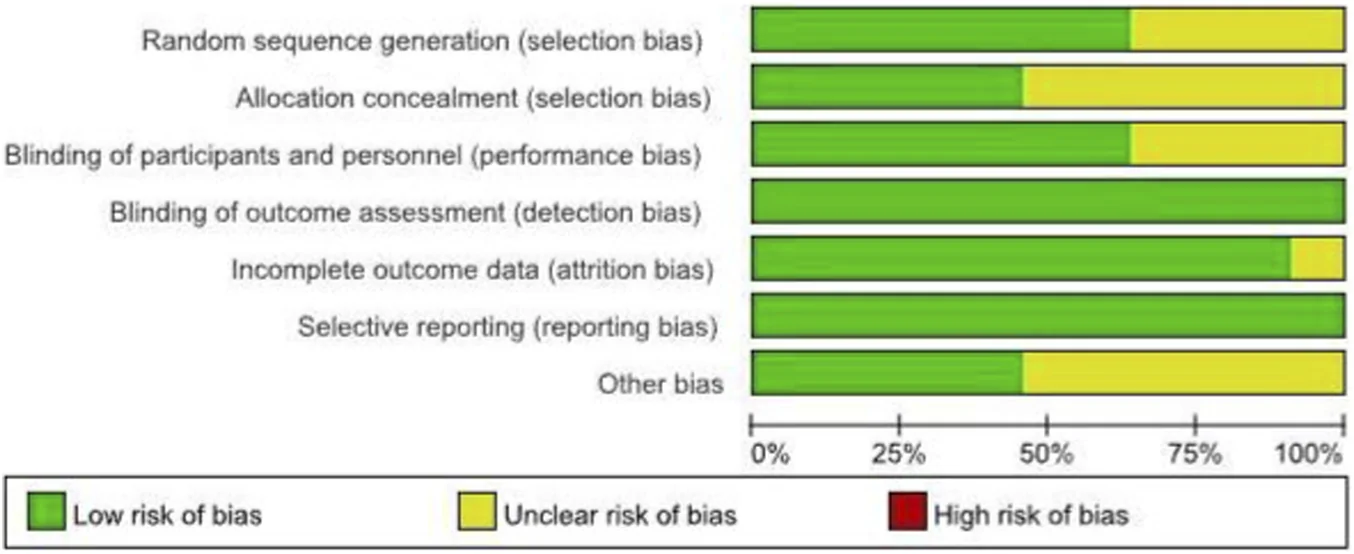
Summary of risk-of-bias assessments across the included studies.

**FIGURE 3:**
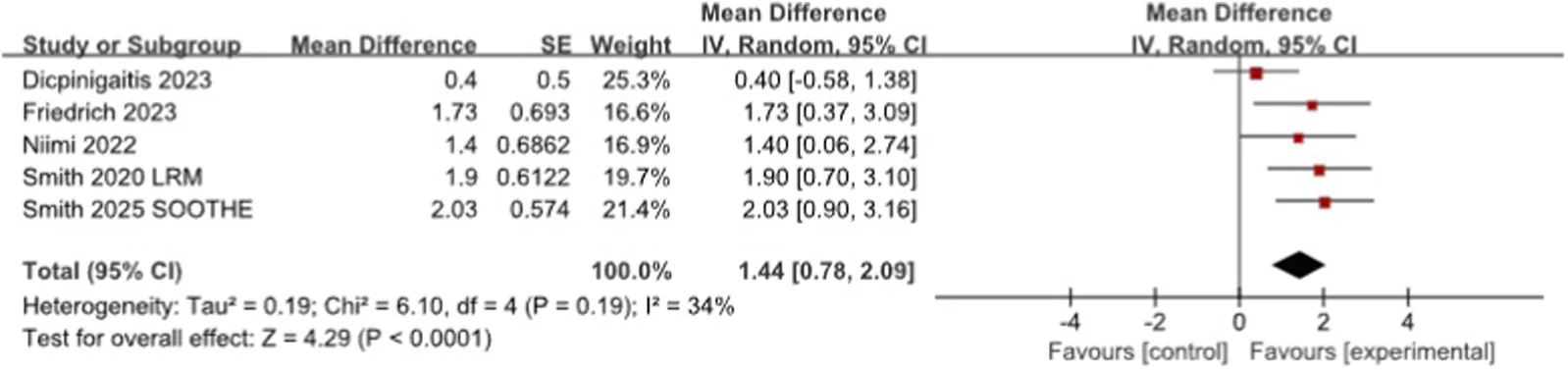
Forest plot of the effect of P2X3 receptor antagonists versus placebo on leicester cough questionnaire (LCQ) total score.

### 3.4 Outcomes of meta-analysis

The pooled estimates for all prespecified efficacy, safety, and tolerability outcomes are summarized in Table 3.

**TABLE 3:** Pooled meta-analysis results.

Outcome	k	Measure	Effect (95% CI)	p	I^2^	Notes
Objective cough frequency	13	Ratio	0.71 (0.63–0.79)	<0.001	57.7%	Relative reduction 29.3%
Taste-related adverse events	11	RR	7.36 (4.84–11.21)	<0.001	42%	Primary tolerability outcome
LCQ total score	5	MD	1.44 (0.78–2.09)	<0.001	34%	Higher score favors treatment
Cough severity VAS	9	MD	−12.73 (−17.10 to −8.36)	<0.001	60%	Negative value favors treatment
Overall adverse events	11	RR	1.21 (1.07–1.38)	0.003	72%	Longest randomized exposure where available
Discontinuation due to AEs	11	RR	3.62 (2.65–4.93)	<0.001	0.0%	Zero-zero trials omitted from RR pooling

#### 3.4.1 Objective cough frequency

Thirteen independent comparisons contributed data to the objective cough-frequency analysis. Compared with placebo, P2×3 receptor antagonists reduced objective cough frequency, with a pooled ratio of 0.71 (95% CI 0.63 to 0.79; *P* < 0.001; *I* ^*2*^ = 57.7%). This corresponded to a 29.3% relative reduction (95% CI 21.2%–36.5%) (Figure 4).

**FIGURE 4:**
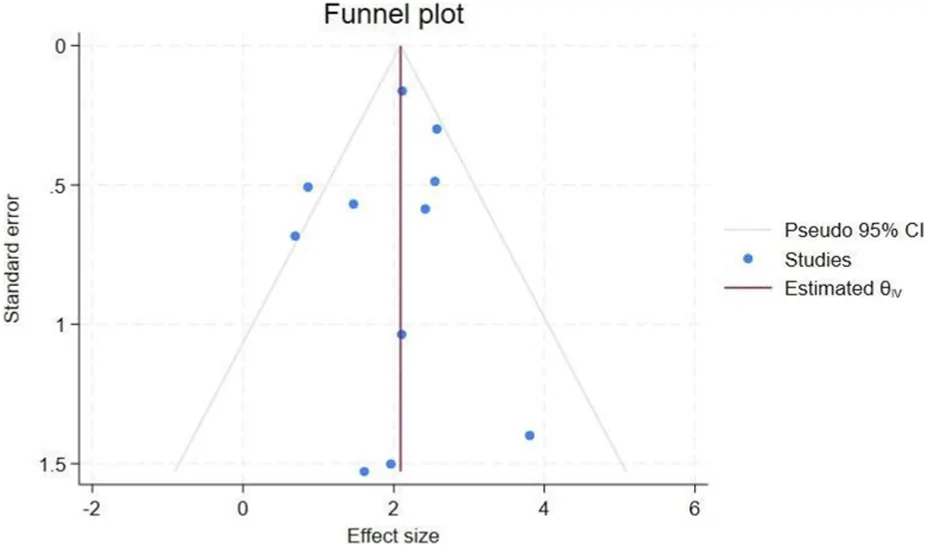
Forest plot for objective cough frequency.

#### 3.4.2 Taste-related adverse events

Taste-related adverse events, as the primary tolerability outcome, were available from 11 comparisons. P2X3 receptor antagonists were associated with a higher risk of taste-related adverse events than placebo (RR 7.36, 95% CI 4.84 to 11.21; *P* < 0.001; *I* ^*2*^ = 42%) (Figure 5).

**FIGURE 5:**
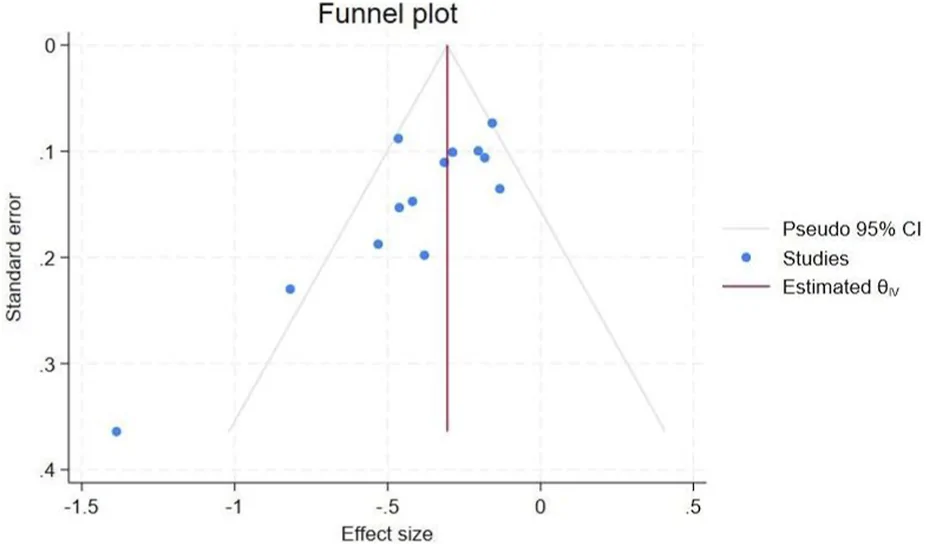
Forest plot for taste-related adverse events.

#### 3.4.3 LCQ total score

Five comparisons reported extractable LCQ treatment differences. The pooled mean difference in LCQ total score was 1.44 points (95% CI 0.78 to 2.09; *P* < 0.001; *I* ^*2*^ = 34%) in favor of P2X3 receptor antagonists (Figure 6).

**FIGURE 6:**
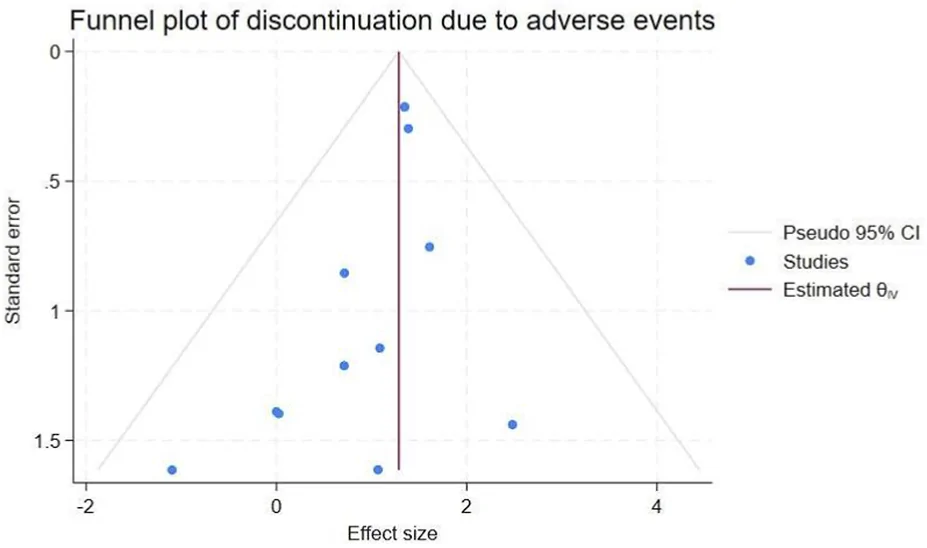
Forest plot of Leicester Cough Questionnaire total score comparing P2X3 receptor antagonists with placebo.

#### 3.4.4 Cough severity VAS

Nine comparisons reported extractable cough severity VAS or CS-VAS data. The pooled mean difference was −12.73 mm (95% CI -17.10 to −8.36; *P* < 0.001; *I* ^*2*^ = 60%) (Figure 7).

**FIGURE 7:**
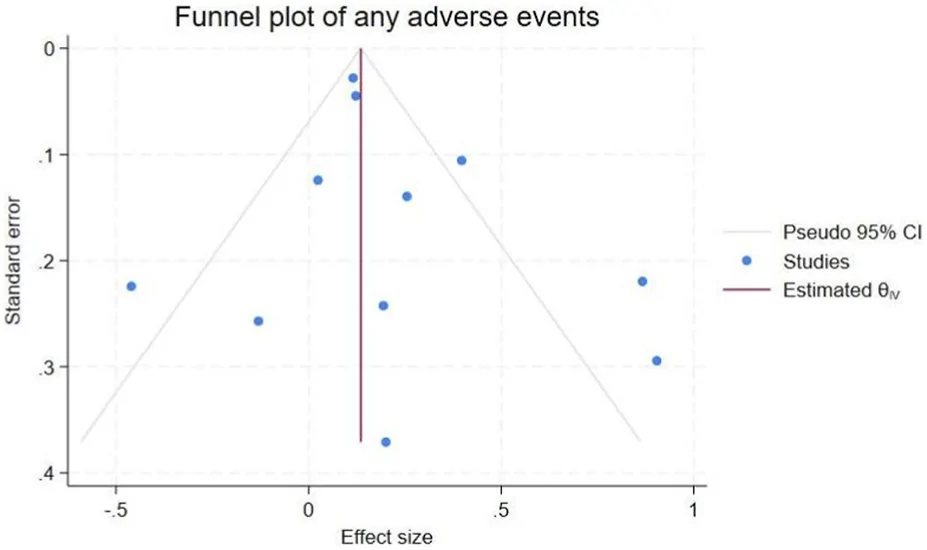
Forest plot of cough severity visual analogue scale score comparing P2X3 receptor antagonists with placebo.

#### 3.4.5 Overall adverse events

Overall adverse events were reported in 11 comparisons. The pooled RR was 1.21 (95% CI 1.07 to 1.38; *P* = 0.003; *I* ^*2*^ = 72%) for P2X3 receptor antagonists compared with placebo (Figure 8).

**FIGURE 8:**
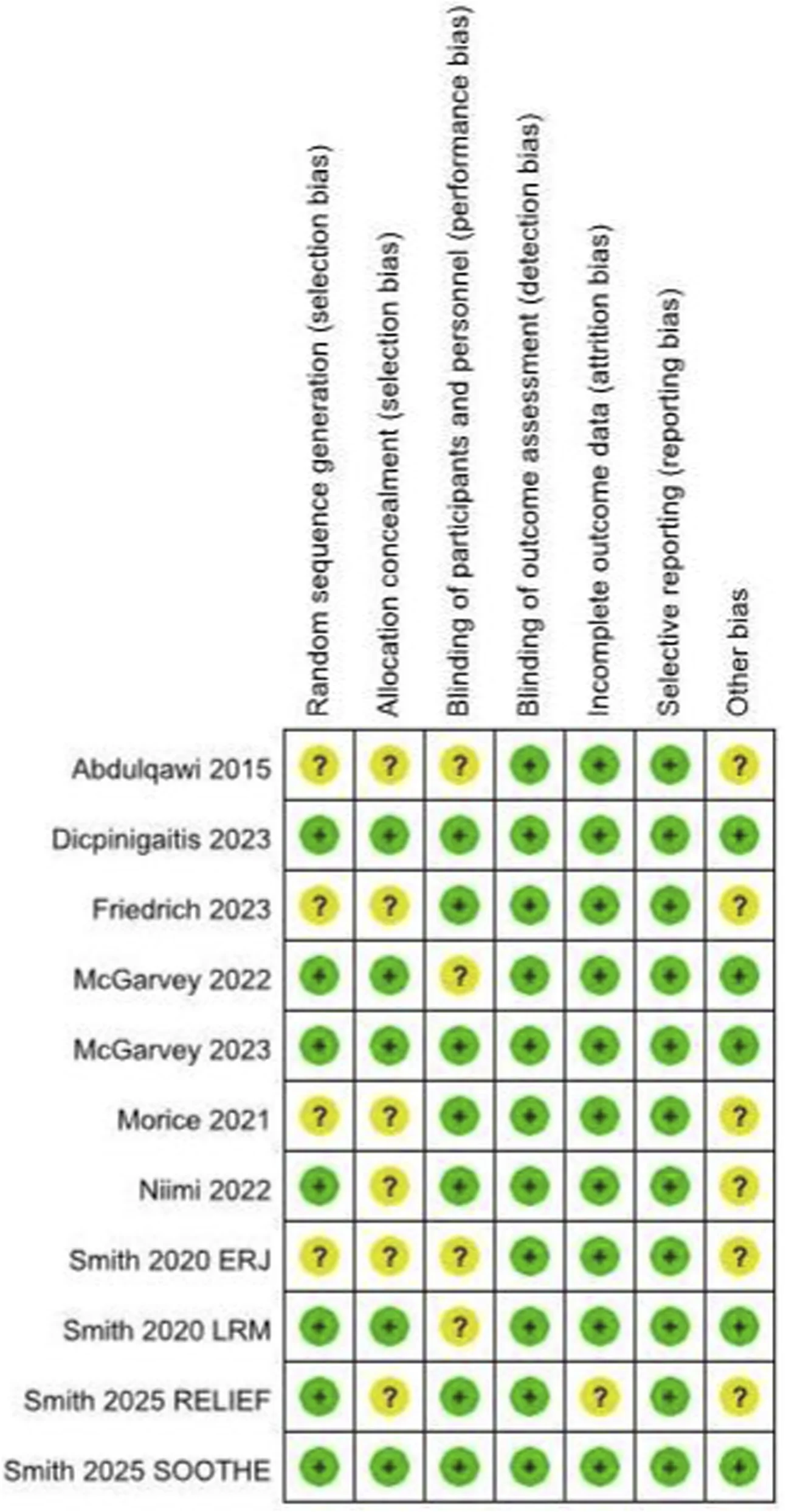
Forest plot of overall adverse events comparing P2X3 receptor antagonists with placebo.

#### 3.4.6 Discontinuation due to adverse events

Discontinuation due to adverse events was reported in 11 comparisons. Discontinuation was more frequent with P2X3 receptor antagonists than with placebo (RR 3.62, 95% CI 2.65 to 4.93; *P* < 0.001; *I* ^*2*^ = 0.0%) (Figure 9).

**FIGURE 9:**
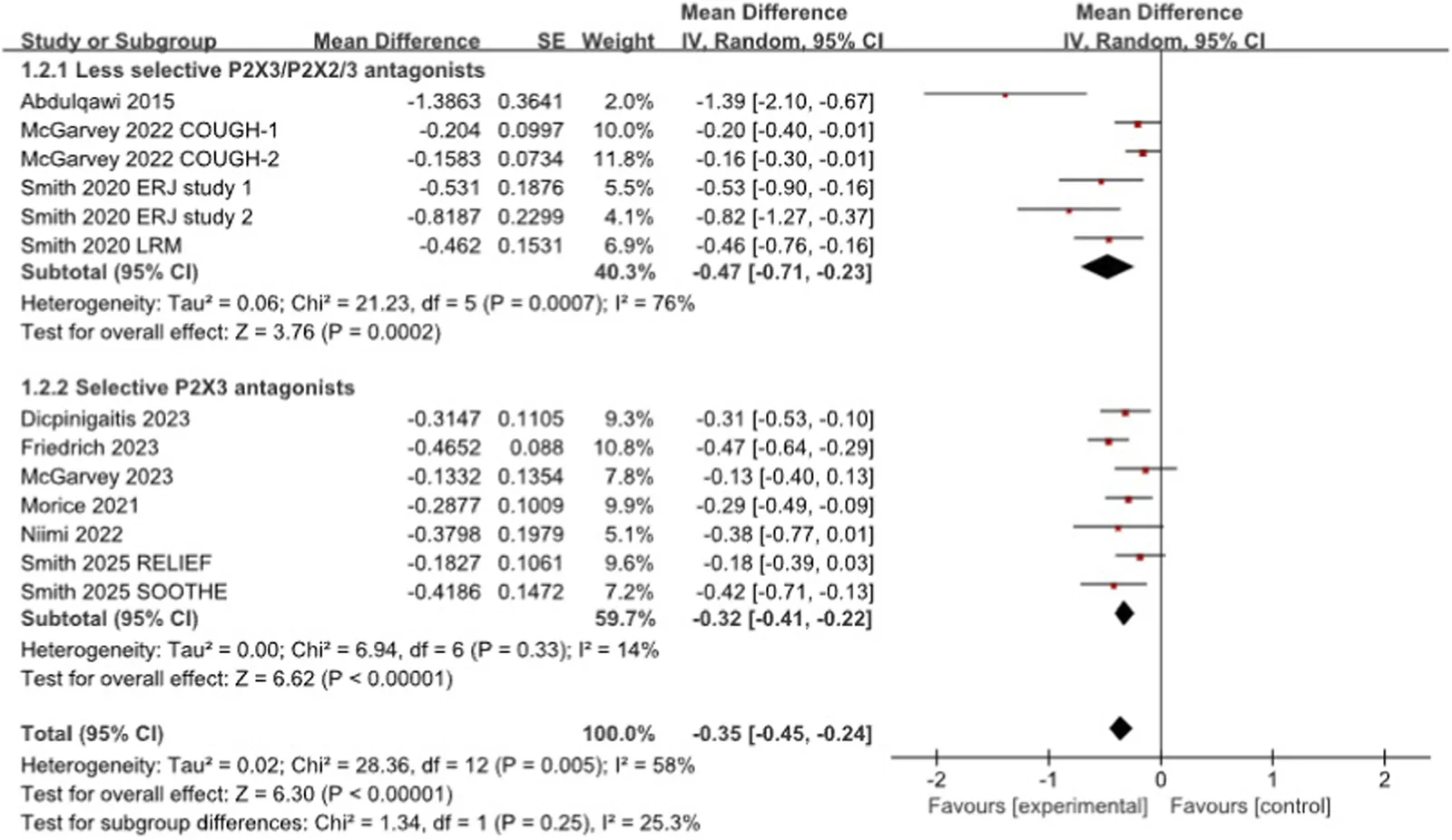
Forest plot of discontinuation due to adverse events comparing P2X3 receptor antagonists with placebo.

### 3.5 Subgroup analyses

Subgroup analyses by receptor selectivity were performed for outcomes with sufficient data. Subgroup estimates were available for objective cough frequency, taste-related adverse events, overall adverse events, and discontinuation due to adverse events. The pooled estimates are presented in Table 4. Potential sources of heterogeneity, including phase of development, treatment duration, study design, and compound-specific receptor selectivity, were considered qualitatively because formal meta-regression would be underpowered and at risk of overfitting. Additional exploratory subgroup analyses were conducted according to trial phase and treatment duration for objective cough frequency and taste-related adverse events. The reduction in objective cough frequency appeared larger in phase 2/early-phase trials than in phase 3 trials, whereas subgroup differences by treatment duration did not reach statistical significance. For taste-related adverse events, increased risks were observed across all phase and duration subgroups, with no statistically significant subgroup differences (Supplementary Figures S1-S4).

**TABLE 4:** Subgroup analysis by receptor selectivity.

Outcome	Subgroup	k	Effect (95% CI)	I^2^
Objective cough frequency	Less selective P2X3/P2X2/3	6	Ratio 0.63 (0.49–0.80)	76%
Objective cough frequency	Selective P2X3	7	Ratio 0.73 (0.66–0.80)	14%
Taste-related adverse events	Less selective P2X3/P2X2/3	4	RR 10.08 (7.17–14.21)	16%
Taste-related adverse events	Selective P2X3	7	RR 4.23 (2.48–7.23)	2%
Overall adverse events	Less selective P2X3/P2X2/3	5	RR 1.24 (1.09–1.41)	72%
Overall adverse events	Selective P2X3	6	RR 1.14 (0.82–1.58)	76%
Discontinuation due to AEs	Less selective P2X3/P2X2/3	6	RR 3.91 (2.83–5.42)	0.0%
Discontinuation due to AEs	Selective P2X3	5	RR 1.66 (0.60–4.59)	0.0%

### 3.6 Sensitivity analysis

Leave-one-out sensitivity analyses showed that no single comparison changed the direction of the pooled estimates. After sequential omission of individual comparisons, the objective cough-frequency ratio ranged from 0.69 to 0.73. The pooled RR ranged from 6.50 to 8.72 for taste-related adverse events, from 1.16 to 1.25 for overall adverse events, and from 3.36 to 3.70 for discontinuation due to adverse events. The pooled MD ranged from 1.18 to 1.81 points for LCQ total score and from −14.19 to −11.50 mm for cough severity VAS. In dose-selection sensitivity analyses using alternative lower efficacy-relevant doses where extractable data were available, the pooled results remained directionally consistent with the main analysis. Objective cough frequency was still reduced with P×23 receptor antagonists (ratio 0.77, 95% CI 0.71 to 0.82; *I* ^*2*^ = 10.1%), and taste-related adverse events remained more frequent than with placebo (RR 6.63, 95% CI 3.88 to 11.32; *I* ^*2*^ = 59.9%) (Supplementary Figures S5 and S6). The leave-one-out results and small-study-effect analyses are summarized in Table 5.

**TABLE 5:** Sensitivity and small-study-effect analyses.

Outcome	Leave-one-out range	Egger p	Begg p	Assessment
Objective cough frequency	Ratio 0.69 to 0.73	0.0004	0.0124	Funnel-plot asymmetry suggested
Taste-related adverse events	RR 6.50 to 8.72	0.8938	0.5334	No statistical evidence of asymmetry
LCQ total score	MD 1.18 to 1.81	Not assessed	Not assessed	k = 5
Cough severity VAS	MD -14.19 to −11.50	Not assessed	Not assessed	k = 9
Overall adverse events	RR 1.16 to 1.25	0.6061	0.6404	No statistical evidence of asymmetry
Discontinuation due to AEs	RR 3.36 to 3.70	0.1954	0.1611	No statistical evidence of asymmetry

### 3.7 Publication bias

Funnel-plot asymmetry and small-study effects were assessed for outcomes with at least 10 comparisons. For objective cough frequency, both Egger’s test (*P* = 0.0004) and Begg’s test (*P* = 0.0124) suggested funnel-plot asymmetry. In contrast, no statistical evidence of small-study effects was detected for taste-related adverse events (Egger *P* = 0.8938; Begg *P* = 0.5334), overall adverse events (Egger *P* = 0.6061; Begg *P* = 0.6404), or discontinuation due to adverse events (Egger *P* = 0.1954; Begg *P* = 0.1611). LCQ total score and cough severity VAS were not assessed statistically because fewer than 10 comparisons were available (Figures 10–13).

**FIGURE 10:**
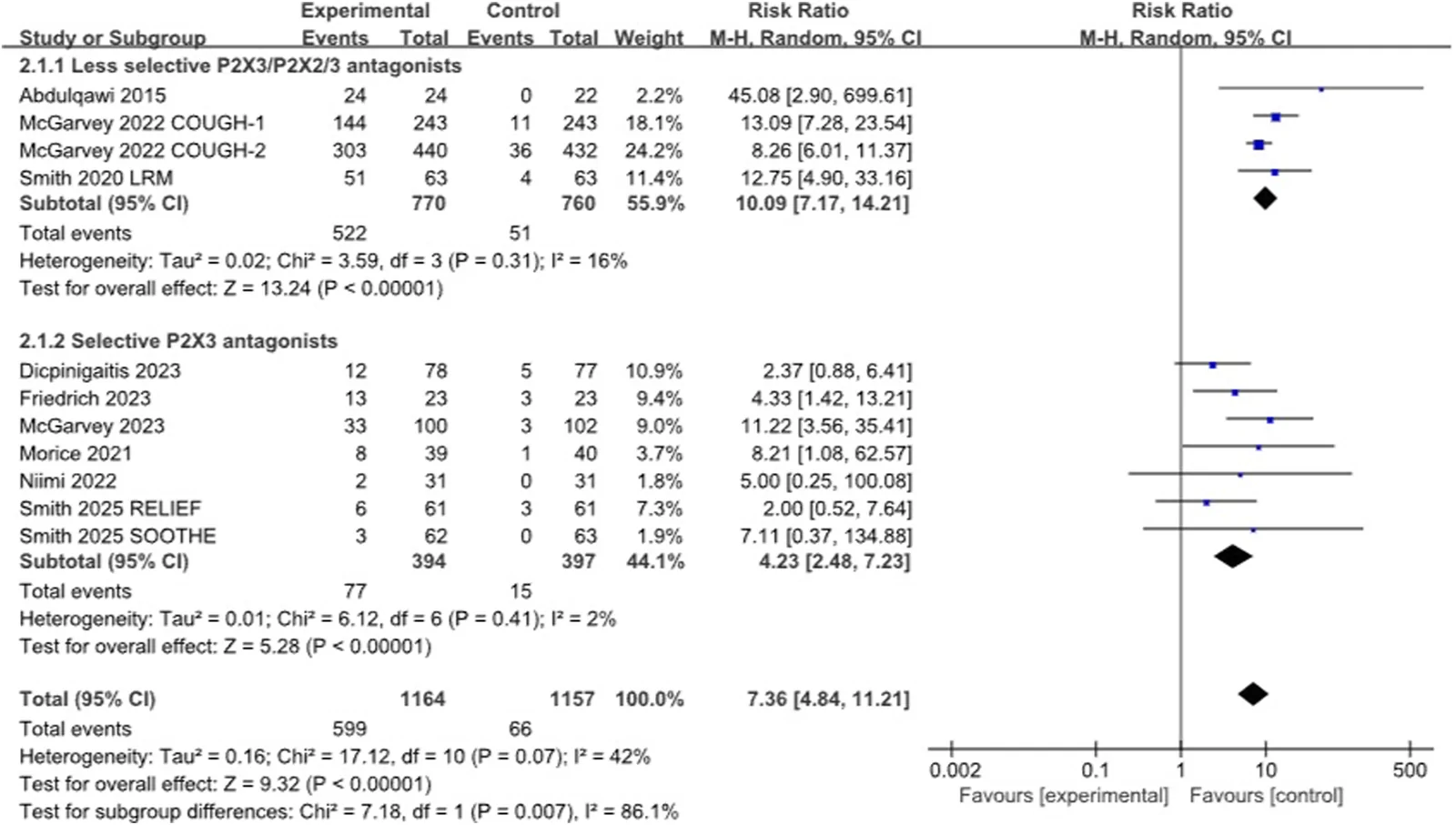
Funnel plot for objective cough frequency.

**FIGURE 11:**
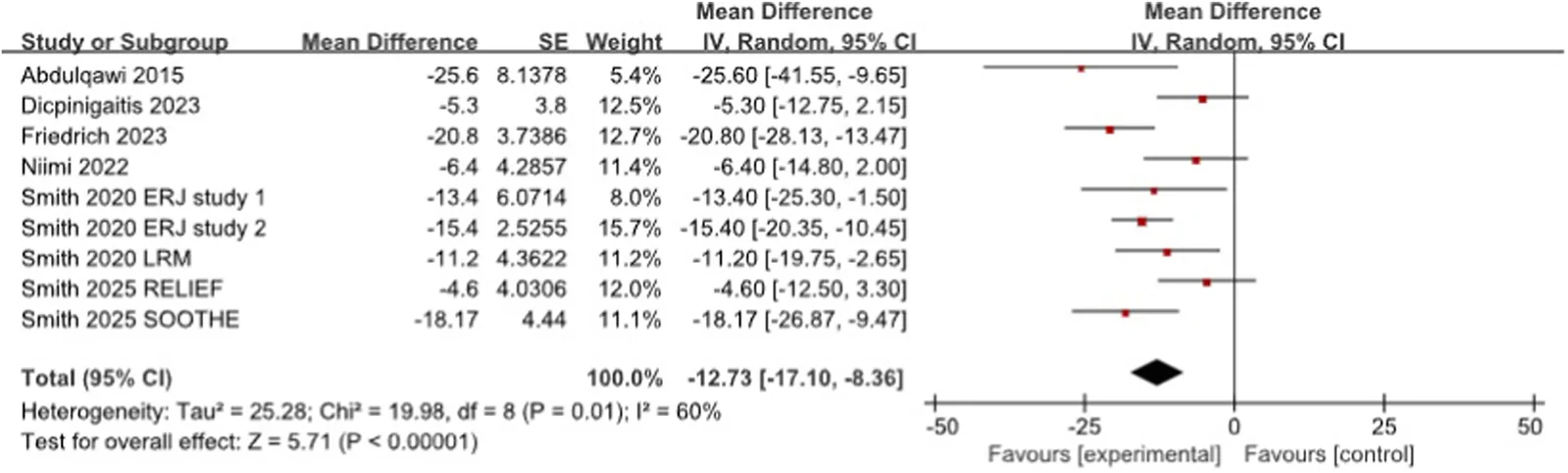
Funnel plot for taste-related adverse events.

**FIGURE 12:**
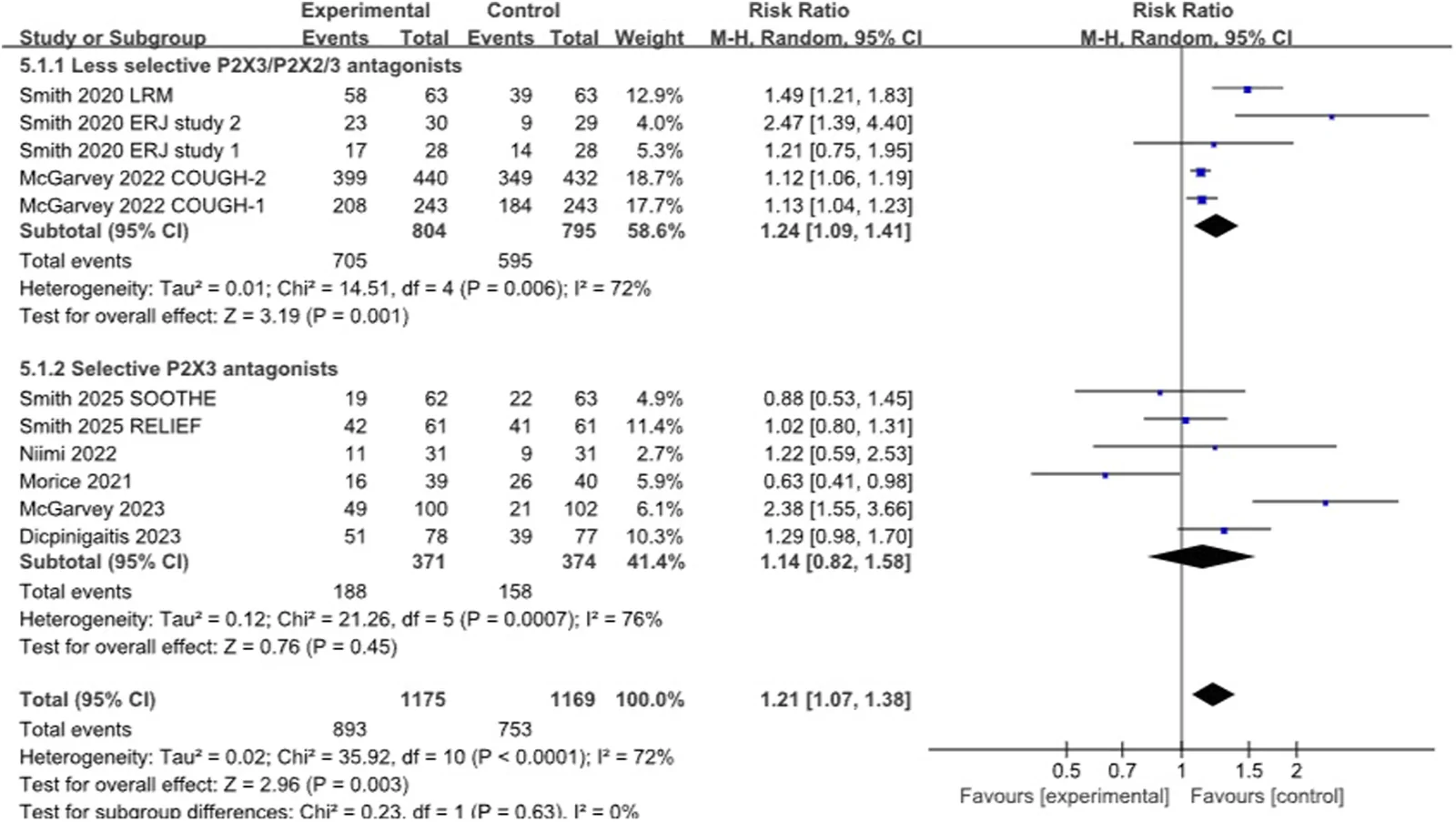
Funnel plot for overall adverse events.

**FIGURE 13:**
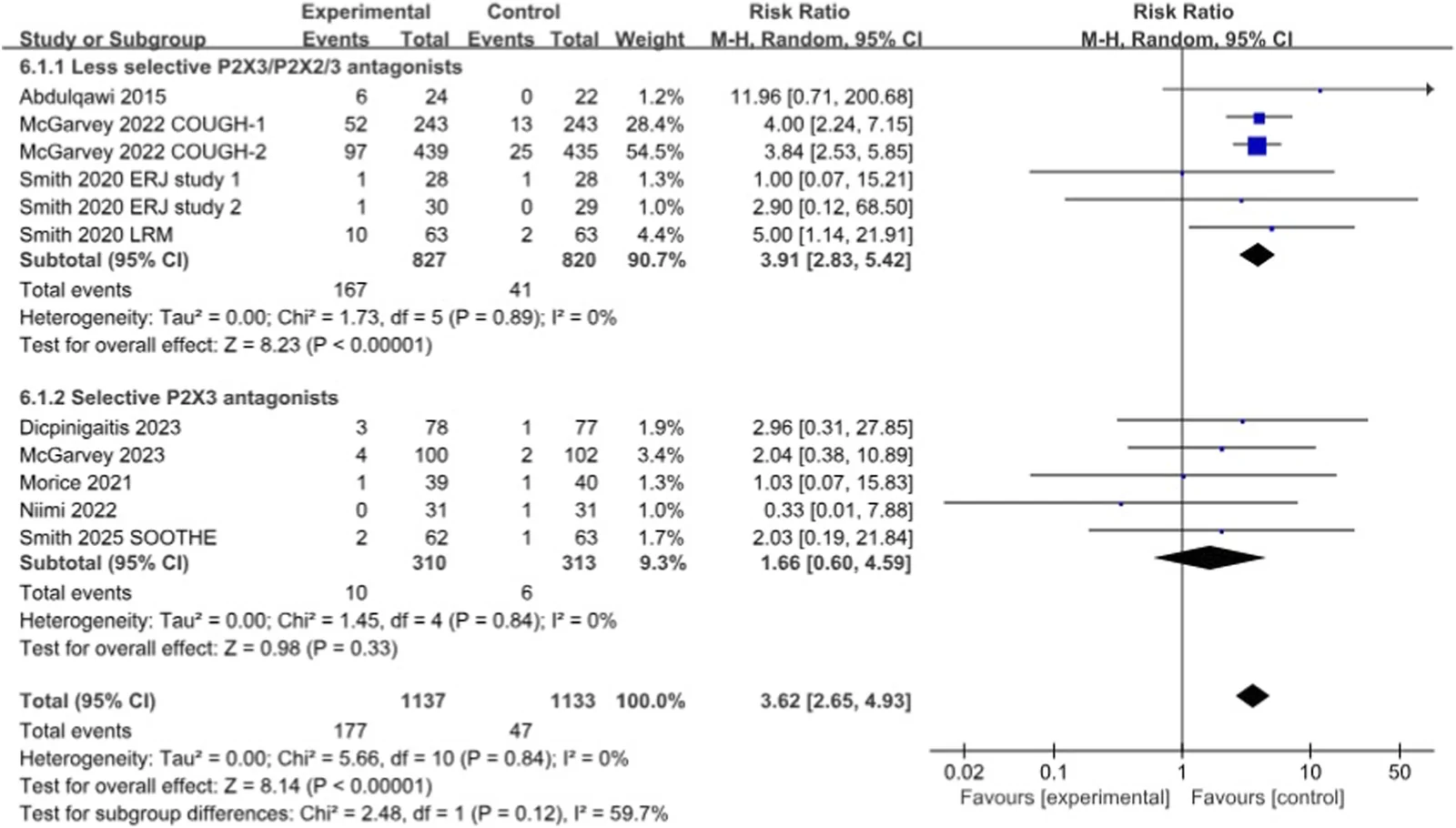
Funnel plot for discontinuation due to adverse events.

### 3.8 Certainty of evidence

Using the GRADE approach, the certainty of evidence was rated as moderate for all prespecified outcomes. The main reasons for downgrading were heterogeneity, possible small-study effects, limited available comparisons, outcome-reporting differences, and possible influence of taste-related adverse events on blinding or treatment discontinuation. The outcome-level GRADE judgments are summarized in Table 6.

**TABLE 6:** GRADE certainty of evidence.

Outcome	Evidence	Effect (95% CI)	Certainty	Main reason
Objective cough frequency	13 comparisons	Ratio 0.71 (0.63–0.79)	Moderate	Downgraded for heterogeneity and possible small-study effects
Taste-related adverse events	11 comparisons	RR 7.36 (4.84–11.21)	Moderate	Downgraded for possible functional unblinding related to taste disturbance
LCQ total score	5 comparisons	MD 1.44 (0.78–2.09)	Moderate	Downgraded for limited comparisons and uncertainty around the minimal important difference
Cough severity VAS	9 comparisons	MD -12.73 (−17.10 to −8.36)	Moderate	Downgraded for substantial heterogeneity
Overall adverse events	11 comparisons	RR 1.21 (1.07–1.38)	Moderate	Downgraded for heterogeneity across drugs and trial durations
Discontinuation due to AEs	11 comparisons	RR 3.62 (2.65–4.93)	Moderate	Downgraded for possible influence of taste-related unblinding on discontinuation decisions

## 4 Discussion

### 4.1 Summary of main findings

In this updated systematic review and meta-analysis of randomized placebo-controlled trials, P2×3 receptor antagonists reduced objective cough frequency in adults with RCC or UCC. The pooled effect corresponded to an approximately 29% relative reduction compared with placebo. Patient-reported outcomes moved in the same direction, with improvement in LCQ total score and reduction in cough severity VAS. Leave-one-out analyses did not change the direction of any pooled estimate, supporting the stability of the main findings. These findings are consistent with previous pairwise and network meta-analyses, while extending the evidence base to include the most recent camlipixant data (Ramadan et al., 2023; Zheng et al., 2023; Yamamoto et al., 2024; Yi et al., 2023).

The safety and tolerability findings were also consistent across the evidence base. Active treatment increased taste-related adverse events, overall adverse events, and discontinuation due to adverse events. The magnitude of the taste-related signal was larger than that of overall adverse events, which suggests that tolerability, rather than general toxicity, is the main tolerability concern for this drug class. Although these events are generally tolerability concerns rather than markers of serious systemic toxicity, they may affect treatment adherence, treatment discontinuation, and maintenance of blinding. The certainty of evidence was rated as moderate for all prespecified outcomes.

### 4.2 Interpretation of efficacy

The reduction in objective cough frequency is clinically meaningful in context because enrolled patients generally had long-standing cough despite prior assessment and treatment of common causes (Morice et al., 2020; Gibson et al., 2016; Satia et al., 2025). The pooled LCQ improvement also approached the commonly used minimal important difference, supporting the view that the physiological effect on cough frequency may translate into patient-perceived benefit (Raj et al., 2009; Trigg et al., 2025). Nevertheless, the average effect should not be interpreted as uniform benefit for every patient. Differences in baseline cough frequency, cough hypersensitivity phenotype, dose, treatment duration, and receptor selectivity may all influence the observed response. Patient-reported outcomes should also be interpreted with some caution because taste disturbance may have led to functional unblinding, whereby participants who experienced dysgeusia, hypogeusia, or ageusia could infer active treatment allocation; this mechanism would be expected to affect LCQ, VAS/CS-VAS, and global impression outcomes more than objectively recorded cough frequency.

The efficacy signal was not confined to gefapixant. Directionally favorable effects were observed across trials of several newer agents, including eliapixant, sivopixant, filapixant, and camlipixant (Morice et al., 2021; Niimi et al., 2022a; Dicpinigaitis et al., 2023; Friedrich et al., 2023; McGarvey et al., 2023a; Smith et al., 2025a; Smith et al., 2025b). This supports P2X3 antagonism as a pharmacological strategy for cough hypersensitivity. At the same time, the magnitude of benefit varied between compounds and doses, and formal dose-response comparisons were beyond the scope of the pairwise main analysis. Additional gefapixant analyses in predefined subgroups, Japanese participants, and patients with recent-onset chronic cough further illustrate that treatment context and patient population may influence both efficacy and tolerability (Smith et al., 2022; Niimi et al., 2022b; McGarvey et al., 2023b).

### 4.3 Receptor selectivity and benefit-risk balance

The subgroup analysis by receptor selectivity is best interpreted as a benefit-risk question. Less selective P2X3/P2X2/3 antagonists showed a numerically larger reduction in objective cough frequency, but they also showed a higher relative risk of taste-related adverse events and a higher risk of discontinuation due to adverse events. Selective P2X3 antagonists showed a smaller pooled effect on cough frequency, but the taste-related tolerability signal was attenuated. The substantial heterogeneity observed for objective cough frequency within the less selective subgroup (I^2^ = 76%) should therefore be interpreted cautiously. This subgroup was dominated by AF-219/gefapixant but combined early crossover dose-escalation studies with later parallel-group trials; differences in selected dose, treatment duration, endpoint timing, baseline cough burden, placebo response, and systemic exposure may all have contributed to the variability.

This pattern is biologically plausible because P2X2/3 heterotrimeric receptors participate in taste signaling, and less selective antagonism may therefore affect gustatory pathways (Finger et al., 2005; Vandenbeuch et al., 2015). However, receptor selectivity should not be presented as a complete solution. Selective P2X3 antagonists still increased taste-related adverse events compared with placebo. The more defensible conclusion is that selectivity may improve the benefit-risk balance, rather than eliminate taste disturbance. Moreover, the selective P2X3 category should not be read as pharmacologically homogeneous: individual compounds differ in measured selectivity, potency, pharmacokinetic profile, and the extent to which nominal doses reflect receptor exposure. Within the selective subgroup, reported P2X3 over P2X2/3 selectivity also differs among agents, and camlipixant, sivopixant, eliapixant, and filapixant should not be assumed to be interchangeable. This subgroup should therefore be viewed as exploratory and mechanism-based rather than as evidence of pharmacological equivalence among individual compounds.

### 4.4 Comparison with previous evidence

Previous systematic reviews and network meta-analyses have reported that P2X3 receptor antagonists reduce cough frequency but increase taste-related adverse events (Ramadan et al., 2023; Zheng et al., 2023; Yamamoto et al., 2024; Yi et al., 2023). The present analysis is consistent with that overall conclusion. Its main contribution is the inclusion of the most recent camlipixant trials and the use of a clinically interpretable subgroup analysis according to receptor selectivity. The results therefore refine, rather than replace, the existing evidence: the class has reproducible antitussive activity, but its place in treatment will depend on whether tolerability can be improved without loss of efficacy.

### 4.5 Strengths and limitations

This review has several strengths. Only randomized placebo-controlled trials were included, which reduced confounding and strengthened the internal validity of the pooled estimates. The analysis incorporated both objective cough-frequency outcomes and patient-reported measures, allowing efficacy to be assessed from complementary perspectives. Safety and tolerability were evaluated separately through taste-related adverse events, overall adverse events, and discontinuation due to adverse events. In addition, the subgroup analysis according to receptor selectivity was clinically relevant, because receptor selectivity is closely related to both the expected pharmacological effect and the main tolerability concern of this drug class.

Several limitations should be considered when interpreting the findings. First, the included trials varied in study design, treatment duration, dose, endpoint timing, and eligibility criteria, which may partly explain the heterogeneity observed in some outcomes. Second, several studies were early-phase or crossover trials, whereas others were larger parallel-group trials; therefore, the pooled estimates should be interpreted as an overall class-level summary rather than as a direct comparison between individual drugs or doses. The included agents also differed in stage of clinical development and current clinical availability, which may vary across countries; therefore, the pooled estimates should be interpreted primarily as evidence supporting the P2X3 therapeutic mechanism and as an overall class-level estimate, rather than as equally applicable to every individual compound. Third, dose-ranging studies required selection of one clinically relevant comparison for the main pairwise analysis to maintain statistical independence of the placebo group. This approach is appropriate for conventional pairwise meta-analysis, but it does not replace a formal dose-response analysis. In addition, nominal dose levels are not directly comparable across P2X3 antagonists because bioavailability, half-life, receptor potency, and receptor occupancy may differ substantially; therefore, the present dose-based comparisons should not be interpreted as exposure-based or pharmacodynamic equivalence. Fourth, the funnel plot and statistical tests suggested asymmetry for objective cough frequency. This finding is compatible with small-study effects, although it may also reflect the larger effects observed in early small crossover and dose-ranging trials rather than publication bias alone. Fifth, outcome reporting was not fully uniform across trials, and some patient-reported outcomes could not be pooled when sufficient variance information was unavailable. Finally, because aggregate rather than individual participant data were used and the number of comparisons was limited, patient-level predictors of response and tolerability and robust meta-regression by phase, duration, dose, or individual compound could not be explored.

### 4.6 Implications for practice and research

For clinical practice, these findings support P2X3 receptor antagonists as a mechanism-based option for adults with RCC or UCC who remain symptomatic after treatable traits have been addressed (Morice et al., 2020; Gibson et al., 2016; Satia et al., 2025; Song et al., 2025; Peters et al., 2025). The decision to use these drugs should be individualized. Patients with severe cough, impaired quality of life, or limited alternative options may accept the risk of taste disturbance in exchange for cough reduction, whereas patients with milder symptoms may place greater weight on tolerability. Selective P2X3 antagonists may offer a more favorable balance between efficacy and taste-related adverse events, but taste disturbance should still be discussed before treatment is started. In practice, class-level efficacy and tolerability estimates should be considered together with the development stage and local availability of each compound. Clinical positioning should also consider compound-specific regulatory status and development stage. Gefapixant has the largest phase 3 evidence base and has progressed furthest in regulatory review and clinical availability across regions, whereas other agents remain investigational or have uncertain development trajectories. After the final search date, GSK reported topline CALM-1 and CALM-2 results for camlipixant: CALM-1 met its primary endpoint with camlipixant 50 mg twice daily, CALM-2 did not reach statistical significance at week 24, the 25 mg twice-daily dose did not meet the primary endpoint in either trial, and GSK stated that it would not progress camlipixant further for RCC (GSK, 2026). Therefore, the pooled results should be viewed as class-level evidence for the P2X3 mechanism rather than as a uniform recommendation for every available or investigational compound.

Future research should focus on three issues. First, dose-finding studies should clarify the lowest effective dose that preserves antitussive efficacy while minimizing taste-related adverse events (Yamamoto et al., 2024; Smith et al., 2022; Niimi et al., 2022b; McGarvey et al., 2023b). Second, trials should use standardized outcome definitions and provide complete variance data for objective cough frequency, LCQ, VAS, and adverse events to facilitate future evidence synthesis (Page et al., 2021; Birring et al., 2008; Birring et al., 2003; Raj et al., 2009; Trigg et al., 2025). Third, longer follow-up and more detailed reporting of baseline characteristics are needed to determine durability of benefit, treatment adherence, and which patients are most likely to respond (Satia et al., 2025; Song et al., 2025; Peters et al., 2025). As more data become available, comparative analyses among selective P2X3 antagonists may help define the optimal place of these agents in chronic cough management.

## 5 Conclusion

P2X3 receptor antagonists were associated with reduced objective cough frequency and improved patient-reported cough outcomes in adults with refractory or unexplained chronic cough. These benefits were accompanied by increased safety and tolerability concerns, particularly taste-related adverse events and discontinuation due to adverse events. Selective P2X3 antagonists may offer a more favorable taste-related tolerability profile than less selective P2X3/P2X2/3 antagonists, but taste disturbance remains a class concern. Further trials should clarify optimal dosing, patient selection, durability of benefit, and long-term tolerability. These findings should be interpreted primarily as class-level evidence rather than as equivalent evidence for every individual compound.

## Data Availability

The original contributions presented in the study are included in the article/Supplementary Material, further inquiries can be directed to the corresponding author.
